# Presumptive treatment with sulphadoxine-pyrimethamine versus weekly chloroquine for malaria prophylaxis in children with sickle cell anaemia in Uganda: a randomized controlled trial

**DOI:** 10.1186/1475-2875-8-237

**Published:** 2009-10-24

**Authors:** Victoria Nakibuuka, Grace Ndeezi, Deborah Nakiboneka, Christopher M Ndugwa, James K Tumwine

**Affiliations:** 1 Department of Paediatrics, Nsambya Hospital, PO Box 7146 Kampala, Uganda; 2 Department of Pediatrics and Child Health, School of Medicine, Makerere University College of Health Sciences, PO Box 7072, Kampala, Uganda

## Abstract

**Background:**

Malaria carries high case fatality among children with sickle cell anaemia. In Uganda, chloroquine is used for prophylaxis in these children despite unacceptably high levels of resistance. Intermittent presumptive treatment with sulphadoxine-pyrimethamine (SP) has shown great potential for reducing prevalence of malaria and anaemia among pregnant women and infants.

**Objective:**

To compare the efficacy of monthly SP presumptive treatment, versus weekly chloroquine for malaria prophylaxis in children attending the Sickle Cell Clinic, Mulago Hospital.

**Methods:**

Two hundred and forty two children with sickle cell anaemia were randomized to presumptive treatment with SP or weekly chloroquine for malaria prophylaxis. Active detection of malaria was made at each weekly visit to the clinic over one month. The primary outcome measure was the proportion of children with one malaria episode at one month follow-up. The secondary outcome measures included malaria-related admissions and adverse effects of the drugs.

**Results:**

Ninety-three percent (114/122) of the children in the chloroquine group and 94% (113/120) in the SP group completed one month follow up. SP reduced prevalence of malaria by 50% compared to chloroquine [OR = 0.50, (95% CI 0.26-0.97)]; p = 0.042. Six percent (7/122) of the children receiving weekly chloroquine had malaria related admissions compared to 2.5% (3/120) on presumptive treatment with SP. No serious drug effects were reported in both treatment groups

**Conclusion:**

Presumptive treatment with SP was more efficacious than weekly chloroquine in reducing prevalence of malaria in children with sickle cell anaemia. Continued use of chloroquine for malaria chemoprophylaxis in children with sickle cell anaemia in Uganda does not seem to be justified.

**Clinical Trials Registration:**

ClinicalTrials.gov Identifier: NCTOO124267

## Background

Sickle cell anaemia (SCA) is a major health problem in Uganda with an average of 25,000 babies born annually [1,2]. Eighty percent of these may die of malaria before two years of age [3]. In Africa, an average of 200,000 babies are born with SCA annually and 50% die before five years of age secondary to anaemia, pneumonia and malaria [3,4].

Persons with SCA are four times more susceptible to malaria than those with sickle cell trait. Malaria is a major contributor to morbidity and mortality in these children [3-7]. It precipitates both anaemia and painful crises and increases the risk of death [4]. In Ghana painful crises occurred frequently during high malaria transmission and malaria accounted for 15.7% of the painful crises requiring admission [8,9].

In Uganda, chloroquine chemoprophylaxis was first used in 1962 where it significantly reduced malaria incidence by 43% [7,10]. While chloroquine resistance was negligible then, in the recent past it has become unacceptably high ranging from 60 to 80% [7,11,12]. Some recent studies show that chemoprophylaxis with chloroquine does not appreciably reduce morbidity due to malaria in children. For example, in Ethiopia, clinic visits for morbidity due to malaria were not reduced by chemoprophylaxis with chloroquine [13]. Another study from Uganda, reported malaria parasitaemia in 44% of sicklers with anaemic crises despite receiving weekly chloroquine[14].

Intermittent presumptive treatment is a new approach to malaria prevention. This strategy was first used among pregnant women among whom it was found to be very effective. It has been successfully used in Tanzania, Ghana and Mozambique among infants [15]. The most successful drug for this strategy is sulphadoxine-pyrimethamine (SP) despite reported resistance to this drug [16]. Hitherto no study been has been carried out in Uganda to assess its efficacy of chloroquine chemoprophylaxis, and yet it remains standard of care for children with sickle cell anaemia. We carried out a double-blind randomized controlled trial to compare the efficacy of weekly chloroquine with monthly SP for malaria prevention in children with sickle cell anaemia in order to inform policy on chloroquine use for chemoprophylaxis.

## Methods

### Patients

The study was carried out at the Sickle cell Clinic situated in Mulago, Uganda's national referral hospital in Kampala, from October 2006 to February 2007. Malaria transmission in this area is mesoendemic and perennial but peaks during the rainy seasons (from March - May, and September - November) [17]. The annual inoculation rate ranges from nine to 10 infective bites per year.

Children between six months and 12 years of age attending the sickle cell clinic were consecutively selected and enrolled if they met the inclusion criteria, that is: 1) living within 10 kilometers from the Hospital; 2) having a negative malaria smear; 3) parents or guardians consented to participation. Children with a history of allergy to sulphonamides were excluded; those on co-trimoxazole prophylaxis or those with any severe illnesses needing admission. The study was approved by Makerere University Faculty of Medicine Ethics and Research Committee and Uganda National Council for Science and Technology.

### Design

This was a double-blind randomized controlled trial, where children between six months and 12 years of age attending the sickle cell clinic were randomized to receive either monthly SP or weekly chloroquine. The sample size of 242 was based on the assumption that the prevalence of malaria would 29.4% in the chloroquine group and 11.1% in the SP group [18,19] with an α = 0.05, a power of 90% and an estimated 10% loss to follow.

### Randomization and blinding

A paediatrician not involved in the study generated a set of 242 random numbers. These numbers were then assigned to two codes in blocks of 4 to 12. Each participant was assigned a serial enrolment number and a random number corresponding to the treatment code. The pills were packed in an opaque envelope labeled with both the study serial number and a random number corresponding to the intervention. The drugs were packed in envelopes and released to the study team only after an eligible participant had been enrolled. The drugs and placebo were similar in colour and shape. They were manufactured by the same company (Kampala Pharmaceutical Industries) and labeled using two codes. Each (CQ) sugar coated tablet contained 150 mg chloroquine base and SP tablet contained sulphadoxine 500 mg and pyrimethamine 25 mg. The treatment code was concealed to the study participants, the treatment nurse and the investigators. The randomization code was only released to the investigators following completion of data analysis.

### Drug administration

The drug were administered by a treatment nurse according to the body weight of the patients as follows: chloroquine 5 mg/kg and sulphadoxine(25 mg/kg)-pyrimethamine (1.25 mg/kg). Participants were observed for 30 minutes after drug administration and treatment re-administered if vomiting occurred.

### Follow up and outcomes

Patients were followed up weekly for one month. At each visit, a finger prick for malaria parasites and parasite density estimation was done, in addition to haemoglobin estimation by Drabkin's method. Parents were requested to report any adverse events to the investigators. A malaria episode was considered if the study participant had fever documented by a temperature of ≥ 37.5°C and presence of any *Plasmodium falciparum *parasite. Children who developed malaria were treated according to the national treatment guidelines at the time (artemether-lumefantrine for uncomplicated malaria, and quinine for severe malaria).

### Statistical analysis

Analysis was by intention to treat: that is all patients randomized were analysed.

The occurrence of at least one episode of malaria in the two treatment groups was compared using Odds Ratios and 95% confidence interval. The student's t-test was used for continuous variables. Logistic regression analysis was used to predict variables affecting the occurrence of malaria.

## Results

### Trial profile (Figure 1)

**Figure 1:**
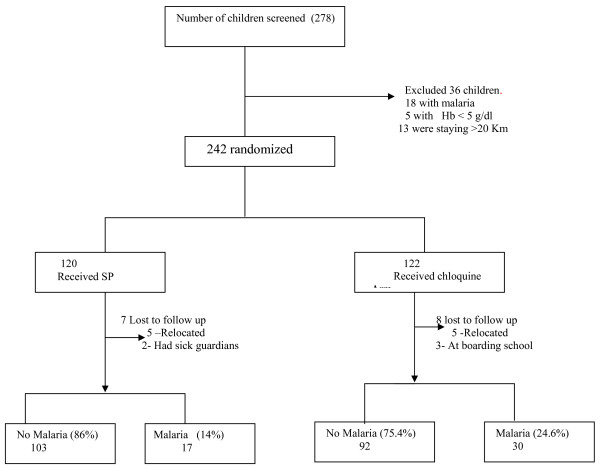
**Trial profile**.

During this period 278 patients were screened and 36 patients did not fulfill the inclusion criteria. Two hundred and forty two were randomized to either SP (monthly) or chloroquine weekly (120 and 122 respectively). Seven patients were lost to follow up from the SP arm and 8 from the CQ arm. All these patients were lost follow up during the first week after enrollment. Five patients from each group that were lost to follow-up relocated to other places during the study period. The rest had different reasons such as a guardian's busy work schedule or having a sick guardian. Two hundred and twenty seven patients were followed up for one month (113 SP arm and 114 CQ arm). Baseline and laboratory characteristics (Table 1) were similar in both treatment groups implying successful randomization.

**Table 1:** Baseline characteristics of patients in both treatment arms at the time of enrollment

**Variable**	**Treatment SP** **N = 120**	**Treatment CQ** **N = 122**	**Odds ratio**	**95% CI**	**P- Value**
**Fever** Yes No	26 (21.7%) 94 (78.3%)	24 (19.7%) 98 (80.3%)	1.129	0.06- 2.105	0.07
**Joint pains** Yes No	40 (38.3%) 80 (73.3%)	49 (40.2%) 73 (59.8%)	0.745	0.441-1.259	0.27
**Headache** Yes No	11 (9.2%) 109 (90.8%)	13 (10.7%) 109 (89.3%)	0.846	0.363-1.971	0.69
**Drugs for malaria prophylaxis before the study** Yes No	99 (48.3%) 21(56.8%)	106 (51.7%) 16 (43.2%)	0.712	0.351-1.441	0.89
**Bed net use** **Yes** **No**	77 (64.2%) 43 (35.8%)	98 (80.3%) 24 (19.7%)	0.44	0.23 -0.82	**0.05**
**Pallor** Yes No	115 (95.8%) 5 (4.2%)	115 (94.3%) 7 (5.7%)	1.4	0.432- 4.540	0.57
**Jaundice** Yes No	110 (91.7%) 10 (90.2%)	110 (90.2%) 12 (9.8%)	1.2	0.49 - 2.893	0.68
**Age (yrs) mean (SD)** Age < 5 yrs Age > 5 yrs	5.49 (4.5) 67 (55.8%) 53 (44.2%)	5.5 (4.3) 60 (49.2%) 62 (50.8%)	N/A ... N/A .... N/A ....	N/A ... N/A ... N/A ...	0.89 0.30
**Sex** Female Male	66(55%) 54(45.0%)	55(45.1%) 67(54.9%)	N/A ... N/A ...	N/A ... N/A ...	0.01*
**Weight (kg)**	18.43 ± 7.7	18.10 ± 6.8	N/A ...	N/A ...	0.72
**Pulse (per min.)**	92 ± 13.8	93 ± 15.0	N/A ...	N/A ...	0.58
**Temperature(°C)**	36.79 ± 2.0	36.5 ± 2.1	N/A ...	N/A ...	0.63

**Haemoglobin (g/dl)**	7.3 ± 1.29	7.2 ± 1.25	N/A ...	N/A ...	0.45

* Values are numbers, percentage unless other wise stated; N/A - Not applicable

### Treatment outcome

#### Proportion of children with malaria

Only 14% (17/120) of the children in the SP arm contracted malaria compared to 24.6% (30/122) in the CQ arm by one month follow-up. Children receiving chloroquine were almost two times more likely to have malaria compared with those receiving SP (Odd's Ratio 1.98 95% CI 1.023 - 3.82).

#### Malaria-related admissions

A higher proportion of children had malaria related admissions in the CQ arm, compared to those in the SP arm (5.7% versus 2.5%). Children receiving chloroquine were almost two and half times more likely to be admitted than those receiving SP. (OR = 2.4, 95% CI 0.6 - 10.0) though this was not statistically significant, p = 0.223.

#### All cause admissions

Reasons for admissions were as follows; SP group: malaria (2), malaria and anaemia (1), anaemia (2) septicaemia and anaemia (1). Chloroquine group: lobar pneumonia (1), malaria and anaemia (2), septicaemia (3), malaria with painful crisis (3), and malaria (2). Even though there were more all cause admissions in the chloroquine group (11/122) 9% than in the SP group (6/120) 5%, the difference was not statistically significant (p = 0.22)

#### Drug side effects

Side effects were assessed for on a weekly basis as children actively reported to the clinic. The proportion of children that vomited after administration of the drugs was 6.6% (SP) versus 11.5% (CQ arm. Pruritus was reported in 1.6% of the children in the SP arm versus 1.8% in the CQ arm. Finally blurring of vision was almost similar in both arms (1.6%% CQ versus 0.8% SP). None of the skin rashes documented were suggestive of a drug reaction. No serious adverse drug reactions were reported in any of the treatment arms (Table 2).

**Table 2:** Side effects documented in both treatment groups over one month follow up

**Symptom**	**Treatment SP** **N = 120**	**Treatment CQ** **N = 122**	**Odd's ratio**	**95% C1**	**P Value**
**Vomiting** Yes No	8 (6.6%) 112 (93.4%)	14(11.5%) 108(88.5%)	0.55	0.12 - 1.36	0.20
**Blurred vision** Yes No	2 (1.6%) 118(98.4%)	1(0.8%) 121 (99.2%)	1.6	0.08 - 6.2	0.66
**Pruritus** Yes No	2(1.6%) 118(98.4%)	3(1.8%) 119(98.2%)	0.6	0.07 - 4.67	0.32
**Skin rash** Yes No	5 (4.1%) 115 (95.9%)	3 (1.8%) 119 (98.2%)	1.74	0.38-8.921	0.44

## Discussion

### Children with at least one malaria episode by one month

Children receiving weekly chloroquine were two times more likely to get malaria compared to those on SP. This shows that SP was more protective, reducing the prevalence of malaria by 50% compared to chloroquine. This difference in efficacy between the two drugs could be explained by the fact that resistance to therapeutic treatment of chloroquine (60%-80%) is higher compared to SP (18% - 25%) in Uganda [12,20].

The other reason is that SP has a longer half life and its terminal elimination phase normally exceeds the minimum parasiticidal concentrations (lowest concentrations that give maximum effect). In contrast this does not occur for chloroquine [21].

While SP may have failed as a treatment drug it is still effective for prophylaxis [22]. Several studies from Ghana, Mozambique and Tanzania have documented its usefulness in prophylaxis and particularly in reducing malaria episodes even in areas where resistance is high [16,22].

In the current study, 24.6% of children receiving chloroquine contracted at least one episode of malaria compared to 40% reported in the Ethiopian study [13]. This is probably due to a relatively lower resistance to chloroquine therapeutic treatment reported in Uganda than that reported from Ethiopia [23,24].

### Malaria-related admissions

In our study, SP reduced malaria related admissions by 50% compared to chloroquine, but this was not statistically significant. This suggests that SP may have a role in reducing malaria related admissions. This is in contrast to results from Ghana, where the reduction in malaria related admissions by SP was 39% in comparison to placebo among children without sickle anaemia[20].

### All cause admissions

Although all cause admissions varied, the percentage of admissions was higher in the chloroquine group (9% versus 5%). Of note, admissions due to lobar pneumonia (1), painful crisis (3) only occurred in those receiving chloroquine. This might be because sulphonamides may offer an anti-bacterial effect against the diseases as has been reported among children with HIV infection [25]. This implies that SP may reduce morbidity from other causes compared with chloroquine. Nonetheless, the reduction in the admissions was not statistically significant and defers from findings reported from Tanzania, and Nigeria [15,26].

### Side-effects

Drug side-effects such as vomiting, skin rash, pruritus, blurred vision were documented in a small proportion of the children (range 0.8% to 11.5%) [15,20]. No serious adverse effects were reported in the two treatment arms and this is consistent with findings in other studies from Tanzania, Mozambique and Ghana [16,22].

## Conclusion

Monthly presumptive treatment with SP was more efficacious than weekly chloroquine in reducing prevalence of malaria among children with sickle cell anaemia. Minor side effects such as pruritus, vomiting, blurred vision and skin rash were reported in a small proportion of patients while no serious adverse events wee documented. Monthly SP should be considered for malaria prophylaxis in children with sickle cell anaemia. Continued use of chloroquine for malaria chemoprophylaxis in children with sickle cell anaemia in Uganda does not seem to be justified.

## Competing interests

The authors declare that they have no competing interests.

## Authors' contributions

VN, GN, CM, DN and JKT designed the study. VN was responsible for patient recruitment. GN, DN, CM and JKT supervised the study. VN, GN and JKT participated in the data analysis and interpretation of the results.

VN, DN and JKT wrote the paper with major contributions of the other authors. All authors have seen and approved the final draft of the manuscript.
